# Supplementary material for: Easy Express Extraction (TripleE)—A Universal, Electricity-Free Nucleic Acid Extraction System for the Lab and the Pen
Source: Microorganisms. 2022 May 23;10(5):1074. doi: 10.3390/microorganisms10051074 (PMC9144652; doi:10.3390/microorganisms10051074)
Supplement: Supplementary file 1 [file microorganisms-10-01074-s001.zip › Table S1_Summary of all four extraction methods.pdf]

Table S1. Summary of all four extraction methods.

Extraction	KingFisher Flex System	IndiMag 48 system	Triple*E* easy-lab	Triple*E* point-of-care
Extraction kit	NucleoMag VET kit (Macherey-Nagel)	IndiMag Pathogen Kit w/o plastic (Indical Bioscience)	IndiMag Pathogen Kit w/o plastic (Indical Bioscience)	IndiMag Pathogen Kit w/o plastic (Indical Bioscience)
Maximal throughput	96	48	8	8
Sample load	100 µl			
Proteinase K	20 µl			
Magnetic beads	20 µl NucleoMag B-Beads	25 µl MagAttract Suspension G	25 µl MagAttract Suspension G	25 µl MagAttract Suspension G
Lysis buffer	100 µl VL1	100 µl VXL	100 µl VXL	100 µl VXL
Binding buffer	350 µl VEB	400 µl ACB	400 µl ACB	400 µl ACB
Wash 1	600 µl VEW 1	700 µl AW1	500 µl AW1	500 µl AW1
Wash 2	600 µl VEW 2	700 µl AW2	500 µl AW1	500 µl AW1
Wash 3	600 µl EOTH 80 %	-	500 µl AW2	500 µl AW2
Wash 4	-	-	500 µl EOTH 80 %	500 µl EOTH 80 %
Elution	100 µl VEL	100 µl AVE	100 µl AVE	100 µl AVE
	Extraction time			
Incubation	5 min	1 min	3 min	1 min
Run	20 min	31 min	7 min	5 min
Total	25 min	32 min	10 min	6 min
